# Evaluation Models for Soil Nutrient Based on Support Vector Machine and Artificial Neural Networks

**DOI:** 10.1155/2014/478569

**Published:** 2014-12-07

**Authors:** Hao Li, Weijia Leng, Yibing Zhou, Fudi Chen, Zhilong Xiu, Dazuo Yang

**Affiliations:** ^1^College of Chemistry, Sichuan University, Chengdu, Sichuan 610064, China; ^2^Key Laboratory of Marine Bio-Resources Restoration and Habitat Reparation in Liaoning Province, Dalian Ocean University, Dalian 116023, China; ^3^College of Life Science and Technology, Dalian University of Technology, Dalian 116021, China

## Abstract

Soil nutrient is an important aspect that contributes to the soil fertility and environmental effects. Traditional evaluation approaches of soil nutrient are quite hard to operate, making great difficulties in practical applications. In this paper, we present a series of comprehensive evaluation models for soil nutrient by using support vector machine (SVM), multiple linear regression (MLR), and artificial neural networks (ANNs), respectively. We took the content of organic matter, total nitrogen, alkali-hydrolysable nitrogen, rapidly available phosphorus, and rapidly available potassium as independent variables, while the evaluation level of soil nutrient content was taken as dependent variable. Results show that the average prediction accuracies of SVM models are 77.87% and 83.00%, respectively, while the general regression neural network (GRNN) model's average prediction accuracy is 92.86%, indicating that SVM and GRNN models can be used effectively to assess the levels of soil nutrient with suitable dependent variables. In practical applications, both SVM and GRNN models can be used for determining the levels of soil nutrient.

## 1. Introduction

### 1.1. Background

Soil nutrient is a crucial property that contributes to the soil fertility and other environment factors [1, 2]. Different components of the soil lead to diverse soil types because of the natural factors, causing various characteristics of the spatiotemporal distribution [3]. According to previous study [4], this variety can make great influence on the regional distribution of vegetation, community biomass, and plant size, as well as the species composition. Therefore, an effective approach is necessary for evaluating the soil nutrient for the sake of scientific management and rational utilization of soil nutrient. Previous research shows that the soil nutrient can be well-estimated by using BP neural networks, principal component analysis, grey relational analysis, fuzzy comprehensive evaluation, and index method [5, 6]. However, these approaches are difficult to operate and the errors are not low enough. Although BP neural networks have a correct result, there still exits a situation that may be not robust enough. Therefore, in this study, we aimed to use support vector machine (SVM), multiple linear regression (MLR), and artificial neural networks (ANNs) for the evaluation of the soil nutrient.

### 1.2. Evaluation Criterion of Soil Nutrient Content

According to the previous study [6], we obtained an admitted criterion of soil nutrient content, which is shown in Table 1.

In this study, we took the content of organic matter, total nitrogen, alkali-hydrolysable nitrogen, rapidly available phosphorus, and rapidly available potassium as independent variables, while the rank of soil nutrient content was taken as dependent variable. The quantized rank of the soil nutrient criterion is the main object to be recognized by models.

### 1.3. Principle of Support Vector Machine

**Figure 1:**
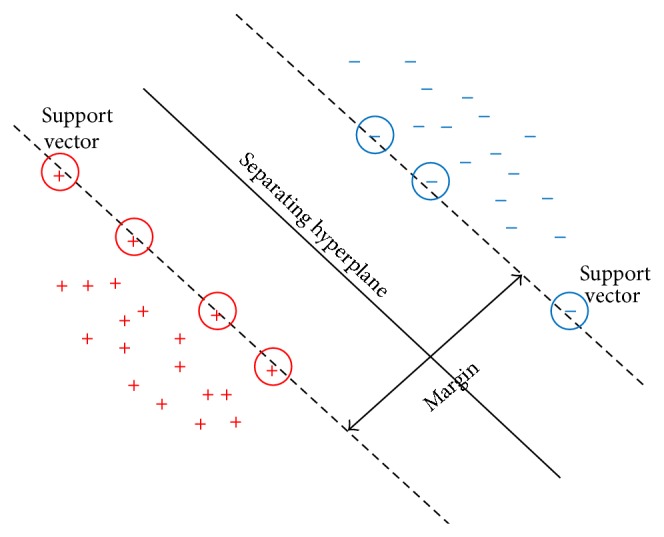
The support vectors determine the position of the optimal hyperplane.

Support vector machine (SVM) is a learning algorithm mainly based on statistical learning theory [7]. On the basis of the limited information of samples between the complexity and learning ability of models, this theory has an excellent capability of global optimization to improve generalization. In regard to linear separable binary classification, finding the optimal hyperplane, a plane that separates all samples with the maximum margin, is an essential principle of SVM. [8, 9]. Not only does the plane help improve the predictive ability of the model, but also it helps reduce the error which occurs occasionally in classifying.  Figure 1 illustrates the optimal hyperplane, with “+” indicating the samples of type 1 and “−” representing the samples of type −1.

**Figure 2:**
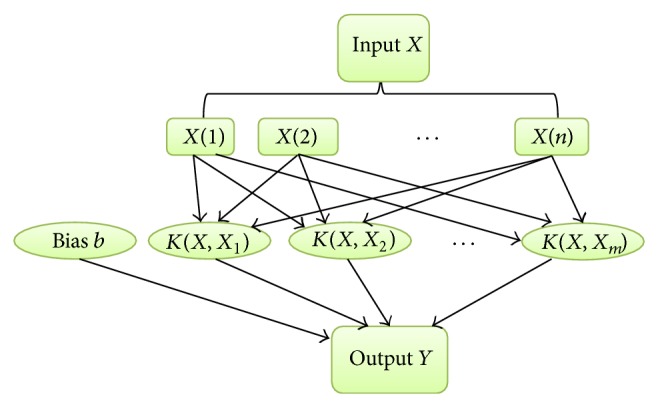
The main structure of support vector machine.

Figure 2 shows the main structure of SVM. The letter “*K*” stands for kernels [10]. As we can see from the figure, it is a small subset extracted from the training data by relevant algorithm that consists of the support vector machine. For classification, choosing suitable kernels and appropriate parameters is of great importance to get a good prediction accuracy. However, a mature international standard currently for us to choose these parameters is nonexistence. In most circumstances, the comparison of experiment result, the experiences from copious calculating, and the use of cross validation that is available in software package are helping us to solve that problem to some extent [11, 12].

### 1.4. Principle of Artificial Neural Networks

An artificial neural network (ANN) model consists of several artificial neurons, which is an adaptive system, equipped to be adapting continuously to new data [13]. It is a powerful tool to deal with nonlinear problems in scientific researches and practical applications, especially in the field of pattern recognition. Structure of the ANN system can be changed in accordance with internal or external information; at the same time, essential data can be extracted from various relevant relationships.

**Figure 3:**
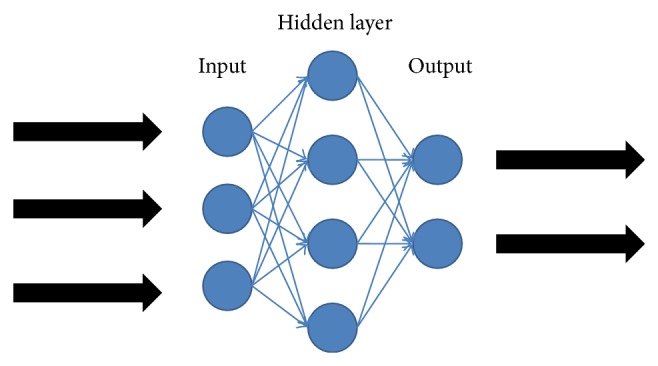
A general structure of artificial neural network.

Figure 3 illustrates the general structure of an artificial neural network (ANN) model. ANN models usually consist of input layers, output layers, and hidden layers. The input variables can be introduced to the network by the input layer [14]. Meanwhile, response variables with predictions, which represent the output of the nodes in this certain layer, are offered by the neural networks. In terms of hidden layers, the type and the complexity of the process determine the optimal number of the neurons in these layers [15]. Our study attempted to use a series of ANN models to classify the rank of soil nutrient quality, which mainly belonged to the application of ANN models in pattern recognition. Besides, we also used multiple linear regression (MLR) for the sake of making comparison, so that the overwhelming advantages of ANN models could be observed.

## 2. Models Development

### 2.1. Support Vector Machine

LIBSVM [16] was applied to construct the multiple classifiers. Cross validation [17] has been applied to the choice of a smoothing parameter of our model to provide a nearly unbiased estimate of classification. In order to compare the prediction accuracy of the SVM model with different proportion of testing and training sets, we randomly chose 35% data as testing set and 65% data as training set in model 1, whereas 20% data as testing set, and 80% data as training set in model 2, respectively. For analyzing the influence of diverse normalization condition to the classification results, we employed 3 different normalization conditions in pretreatment in each model separately: pretreating the dataset without normalization, normalizing the dataset from −1 to 1 as well as 0 to 1. Each model with different normalization condition was computed 1000 times so as to get the exactly accurate predicting results. Besides, the selection of training and testing sets in every count was random. The average prediction accuracy was also calculated. In this paper, the model whose mean accuracy is relatively high was identified as the optimum.  Table 2 illustrates the results of our computation of different model with diverse normalization condition.

According to Table 2, both the normalization condition and the proportion of the testing set and training set lead to the different prediction accuracy of the soil classification. In this paper, model 2 was calculated without normalization condition which has the highest average prediction accuracy with a relatively lower deviation error considered the optimal with model to estimate the soil nutrient.

### 2.2. Artificial Neural Networks

For comparison, 13 models were established in our experiments, including multiple linear regression model (MLR), general regression neural network (GRNN) [18], and multilayer feed-forward neural network (MLFN) [19, 20]. In these computations, MLR model was used for comparison. And nodes of MLFN models were set to be in the range from 2 to 12; hence the most robust MLFN model could be found. All the models were trained over 100 times, and every time the component of the training set and testing set are different. Afterwards, RMS error and training time of each model were calculated. Results are shown in Table 3.

According to Table 3, the GRNN model is considered as the best ANN model in the evaluation, with an RMS error 0.27. And the average prediction accuracy of GRNN model is 92.86%, slightly higher than those of SVM models. From the results presented in the table, we can see that in terms of the values of RMS error and the training time, the GRNN model still predominates among the ANN models during this experiment. Therefore, we can draw a conclusion that GRNN model is the best ANN model in evaluating soil nutrient.

## 3. Results and Discussion

### 3.1. Training and Testing Results of SVM Model

**Figure 4:**
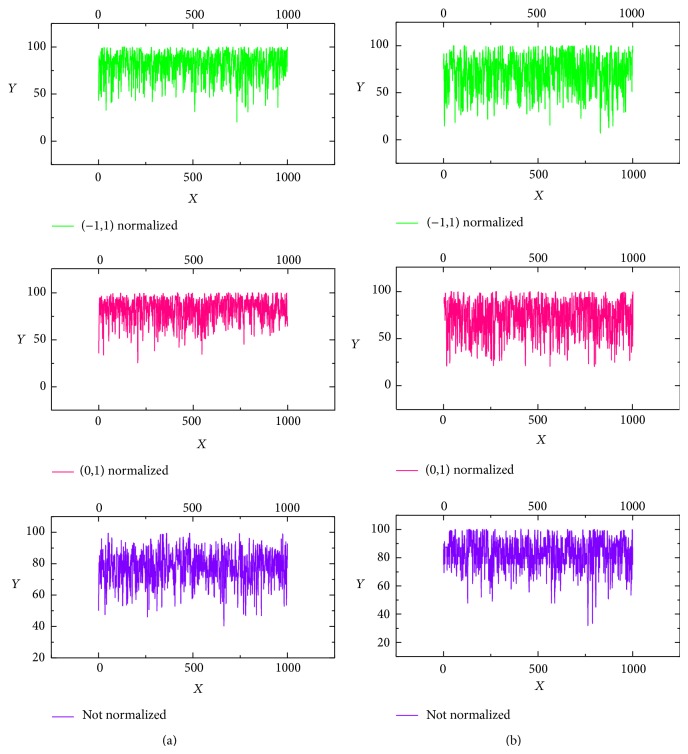
Results of SVM model: (a) different results calculated from diverse normalization conditions in model 1; (b) different results calculated from diverse normalization conditions in model 2.

Figure 4 indicates the fluctuations of the prediction accuracy calculated in diverse normalization condition. We computed each model for 1000 times, axis *Y* represents the value of the prediction accuracy of each count, whereas axis *X* just stands for the time. That is to say, the first point of the picture is the prediction accuracy calculated for the first time, and the second point represents the second results. The last point is the value of the prediction calculation in the last calculation. The fluctuation of the prediction accuracy illustrates the steady level of the model. Color pink represents the data that is normalized in (0, 1), color green stands for the data that is pretreated under the normalization condition (−1, 1), and color violet is the result calculated from the data without normalization.

The training and testing results of the two SVM models presented by Figure 4 show that SVM models can correctly distinguish the ranks of soil nutrient content. Interestingly, it is particularly noticeable that the fluctuation in picture (a) is relatively lower than that in picture (b). We consider that there may be some disruptions caused by the original SVM algorithm. In our further study, we will pay attention to find out the best SVM model on this application of pattern recognition.

### 3.2. Training and Testing Results of ANN Model

According to the comparison results provided in Section 2.2, GRNN model is considered as the most suitable ANN model for evaluation, with the lowest RMS error.

**Figure 5:**
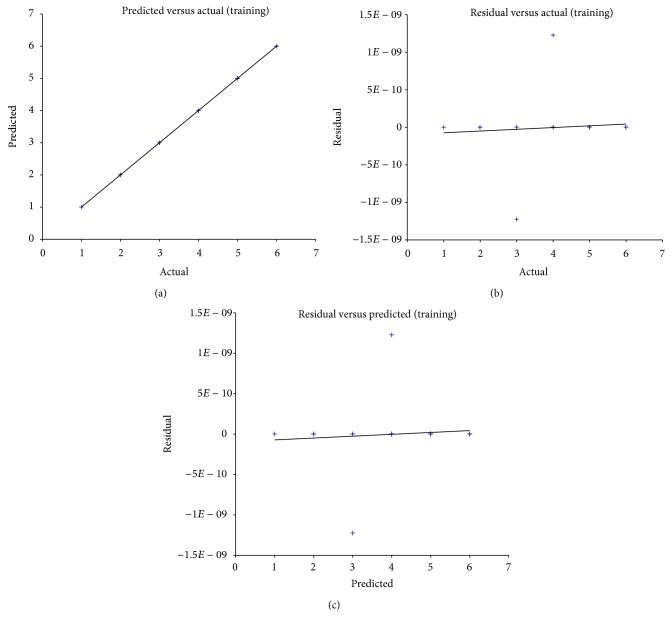
Training results of GRNN model. (a) Comparison between predicted values and actual values, (b) comparison between residual values and actual values, and (c) comparison between residual values and predicted values.

Figure 5 depicts the average training results of GRNN model. Results correspond with the regular training results of artificial neural networks, showing the robust training results of GRNN.

**Figure 6:**
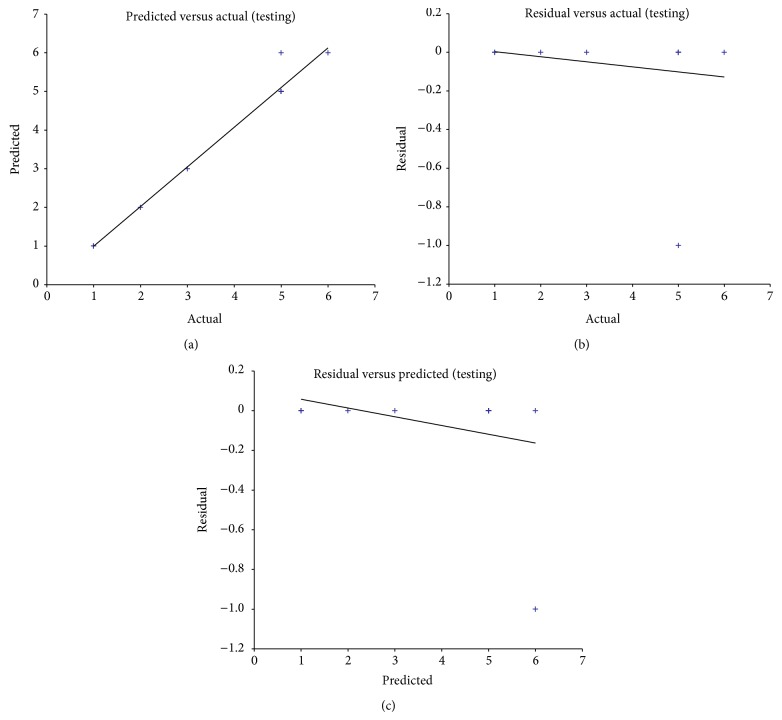
Testing results of GRNN model. (a) Comparison between predicted values and actual values, (b) comparison between residual values and actual values, and (c) comparison between residual values and predicted values.

Figure 6 shows the average testing results of GRNN model. Data are quite concentrated and met the norm results of artificial neural networks, which is the accurate result of GRNN model.

Results of GRNN model's training and testing suggest that ANN model can be used for determining the levels of soil quality. Compared to the SVM models, GRNN model seems more robust and its results are more precise.

### 3.3. Comparison with Previous Studies

We cited several typical researches for making comparison [21–26]. Karlen et al. [21] summarized deliberation by the Soil Science Society of America (SSSA) Ad Hoc Committee on Soil Quality, showing the concept, definition, and framework for evaluation of soil quality. Fox and Kamprath [22] utilized phosphate sorption isotherms for evaluating the phosphate requirements of soils. Binkley and Vitousek [23] discussed the conceptual basis for measuring nutrient availability and describe the strengths and limitations of some of the methods for assessing nonagricultural soils. They also discussed methods for characterizing soil acidity, salinity, and redox potential because they controlled nutrient cycling and availability. Doran and Parkin [24] defined and assessed the evaluation approaches of soil quality, providing us with available methods of soil assessments. Albergel et al. [25] used a series of comprehensive models to evaluate the soil moisture product. Rossel et al. [26] used vis-NIR spectroscopy to determine the soil color, mineral composition, and clay content. These previous studies offer us various experiences to current research; however, there are still no relevant researches on soil evaluation by using SVM, MLR, and ANN methods. Our research can effectively fill the blank of similar areas and it can be applied into more practical applications.

## 4. Conclusions

In this research, we used support vector machine (SVM), multiple linear regression (MLR), and artificial neural networks (ANNs), respectively, to evaluate the soil nutrition. Results show that the average prediction accuracies of SVM models are 77.87% and 83.00%, respectively, while the GRNN's average prediction accuracy is 92.86%, which suggest that SVM and GRNN models can be used to assess the soil nutrient with suitable dependent variables effectively, and GRNN models have a good result, with a low RMS error (0.27). In practical applications, both SVM and GRNN models can be used for determining the levels of soil nutrient.

## Figures and Tables

**Table 1:** The evaluation criterion of soil nutrient content.

Rank	Organic matter/g*·*kg^−1^	Total nitrogen/g*·*kg^−1^	Alkali-hydrolysable nitrogen/mg*·*kg^−1^	Rapidly available phosphorus/mg*·*kg^−1^	Rapidly available potassium/mg*·*kg^−1^
1	>40	>2.0	>150	>40	>200
2	30–40	30–40	120–150	20–40	150–200
3	20–30	20–30	90–120	10–20	100–150
4	10–20	10–20	60–90	5–10	50–100
5	6–10	6–10	30–60	3–5	30–50
6	<6	<0.5	<30	<3	<30

**Table 2:** Computing results of the two models in different normalization conditions.

Model (trained for 1000 times)	Proportion of training set	Computing results	No normalization	[−1, 1] Normalization	[0, 1] Normalization
Model 1	35%	Average prediction accuracy	77.87%	80.91%	82.34%
		Standard deviation	0.1396	0.1911	0.1727

Model 2	20%	Average prediction accuracy	83.00%	71.53%	72.75%
		Standard deviation	0.1463	0.2548	0.2464

**Table 3:** Results of multiple linear regression and artificial neural network models.

ANN model	Trained samples	Tested samples	Average RMS error	Training time	Finishing reason
Linear predictor	27	14	0.53	0:00:00	Auto-stopped
GRNN	27	14	0.27	0:00:00	Auto-stopped
MLFN 2 nodes	27	14	1.03	0:00:35	Auto-stopped
MLFN 3 nodes	27	14	1.58	0:01:07	Auto-stopped
MLFN 4 nodes	27	14	0.69	0:00:58	Auto-stopped
MLFN 5 nodes	27	14	0.38	0:00:38	Auto-stopped
MLFN 6 nodes	27	14	0.36	0:01:01	Auto-stopped
MLFN 7 nodes	27	14	0.50	0:01:19	Auto-stopped
MLFN 8 nodes	27	14	0.35	0:01:31	Auto-stopped
MLFN 9 nodes	27	14	1.48	0:01:48	Auto-stopped
MLFN 10 nodes	27	14	0.46	0:01:58	Auto-stopped
MLFN 11 nodes	27	14	0.38	0:02:22	Auto-stopped
MLFN 12 nodes	27	14	0.50	0:02:57	Auto-stopped
